# The Landscape of Gene Expression and Molecular Regulation Following Spinal Cord Hemisection in Rats

**DOI:** 10.3389/fnmol.2019.00287

**Published:** 2019-11-22

**Authors:** Bin Yu, Chun Yao, Yongjun Wang, Susu Mao, Yaxian Wang, Ronghua Wu, Wei Feng, Yanping Chen, Jian Yang, Chengbin Xue, Dong Liu, Fei Ding, Xiaosong Gu

**Affiliations:** ^1^Key Laboratory of Neuroregeneration of Jiangsu and Ministry of Education, Co-innovation Center of Neuroregeneration, Nantong University, Nantong, China; ^2^Jiangsu Clinical Medicine Center of Tissue Engineering and Nerve Injury Repair, Affiliated Hospital of Nantong University, Nantong University, Nantong, China

**Keywords:** spinal cord injury, RNA-sequencing, microenvironment, astrocyte, microglia, oligodendrocyte, vasculation

## Abstract

Spinal cord injury (SCI) is a challenging clinical problem worldwide. The cellular state and molecular expression in spinal cord tissue after injury are extremely complex and closely related to functional recovery. However, the spatial and temporal changes of gene expression and regulation in various cell types after SCI are still unclear. Here, we collected the rostral and caudal regions to the lesion at 11 time points over a period of 28 days after rat hemisection SCI. Combining whole-transcriptome sequencing and bioinformatic analysis, we identified differentially expressed genes (DEGs) between spinal cord tissue from injured and sham-operated animals. Significantly altered biological processes were enriched from DEGs in astrocytes, microglia, oligodendrocytes, immune cells, and vascular systems after SCI. We then identified dynamic trends in these processes using the average expression profiles of DEGs. Gene expression and regulatory networks for selected biological processes were also constructed to illustrate the complicate difference between rostral and caudal tissues. Finally, we validated the expressions of some key genes from these networks, including α-synuclein, heme oxygenase 1, bone morphogenetic protein 2, activating transcription factor 3, and leukemia inhibitory factor. Collectively, we provided a comprehensive network of gene expression and regulation to shed light on the molecular characteristics of critical biological processes that occur after SCI, which will broaden the understanding of SCI and facilitate clinical therapeutics for SCI.

## Introduction

Spinal cord injury (SCI) causes devastating neurological deficits and disability with 10s of 1000s of new cases around the world every year (Singh et al., 2014). Despite considerable global research efforts, there are currently no effective and reliable clinical treatments for patients with SCI (Silva et al., 2014). In addition, outcomes from standard surgical and pharmacological interventions are inconsistent.

Permanent functional deficits after SCI mainly arise from the weak regeneration capacity of neurons and the inhibitory environment around the injury site (Du et al., 2015). The primary (mechanical) injury—which usually involves contusion and compression by blunt force—results in damage to neurons, axons, and glia at the site of the injury. Over the following days to weeks, the mechanical injury leads to a cascade of biological events within and around the primary lesion, which are collectively referred to as secondary injury. These events include ischemia, edema, inflammation, cellular necrosis, and vascular changes (Silva et al., 2014). Neuronal cell death contributes to SCI-induced neurological deficits; however, much of this is delayed cell death as a result of the secondary injuries rather than direct mechanical damage (Liu et al., 2015).

Numerous biochemical mechanisms have been proposed to explain the progressive lesions in spinal cord tissue after SCI, including apoptosis of neurons and glial cells, inflammatory responses, vascular changes, free radical formation, lipid peroxidation, changes in ionic balance, glutamate excitotoxicity, and glial scar formation (Silva et al., 2014). With respect to the latter, astrocytes transform into a reactive state after SCI, becoming hypertrophic and proliferative, and form a border between the injury site and surrounding tissues. Eventually the reactive astrocytes, microglia, macrophages, and extracellular matrix molecules form a glial scar, which acts as a physical barrier to re-growing axons.

Because of the many processes involved in secondary injuries, an in-depth exploration of the temporal and spatial changes in gene expression and molecular regulation following SCI is of the utmost importance – an understanding of these processes would facilitate the development of novel and effective therapies to minimize the extent of the lesion and improve recovery. A number of previous studies have investigated the changes in molecules, cells, and tissues that occur after SCI (Nishimura et al., 2014; Duan et al., 2015; Shi et al., 2017).

Here, we used a partial spinal transection (hemisection), which is a commonly used animal model of SCI (Kozuka et al., 2016; Martini et al., 2016), and compared changes in the rostral (R) and caudal (C) regions of the injury site with each other and with sham-injured animals over a period of 28 days after injury. We analyzed the dynamic trends in gene expression and the related biological processes using ingenuity pathway analysis (IPA). The spatial and temporal characteristics of key genes in specific cell types and certain biological processes after SCI were determined with subsequent immunofluorescent verification. Overall, our study provides new insights into the cellular and molecular processes after SCI.

## Results

### Differential Gene Expression in Rostral and Caudal Sites Following Spinal Cord Injury

We performed a spinal cord hemisection in rats and collected two 5-mm-long segments from the injury site, one in the rostral (R) direction from the injury and one in the caudal (C) direction (Supplementary Figure S1a). The segments were collected from six rats at each time point after SCI or sham surgery (0, 0.5, 3, 6, 12 h and 1, 3, 7, 14, 21, 28 days) and were subsequently prepared for RNA extraction and RNA sequencing. The map-to-gene and map-to-genome data suggested good sequence quality (Supplementary Table S1).

By comparing the injured (R and C) groups with the sham group (S), we identified differentially expressed genes (DEGs) for both the rostral and caudal regions after SCI. As shown in Supplementary Figure S1b, during the initial period after SCI (0–3 h), there were no significant differences in gene expression between the R, C, and S groups. In the early phase (6 h – 1 day), the number of DEGs increased to 100s in the R and C regions, with no notable differences between these groups. In the middle and late periods (3–28 days), the number of DEGs rapidly increased to 1000s for both the R and C regions. However, the number of DEGs was markedly smaller in the C region than in the R region.

### Molecular Regulation of Astrocyte Responses

Approximately 90% of cells in the CNS are glia, and the majority of these are astrocytes (Ge et al., 2007). After SCI, astrocytes become reactive, with enlarged soma and increased glial fibrillary acidic protein (GFAP) expression. This is referred to as the hypertrophic phase. Hyperplastic responses are followed by an increase in the number of astrocytes, which contributes to glial scar formation (Bhalala et al., 2012).

**FIGURE 1:**
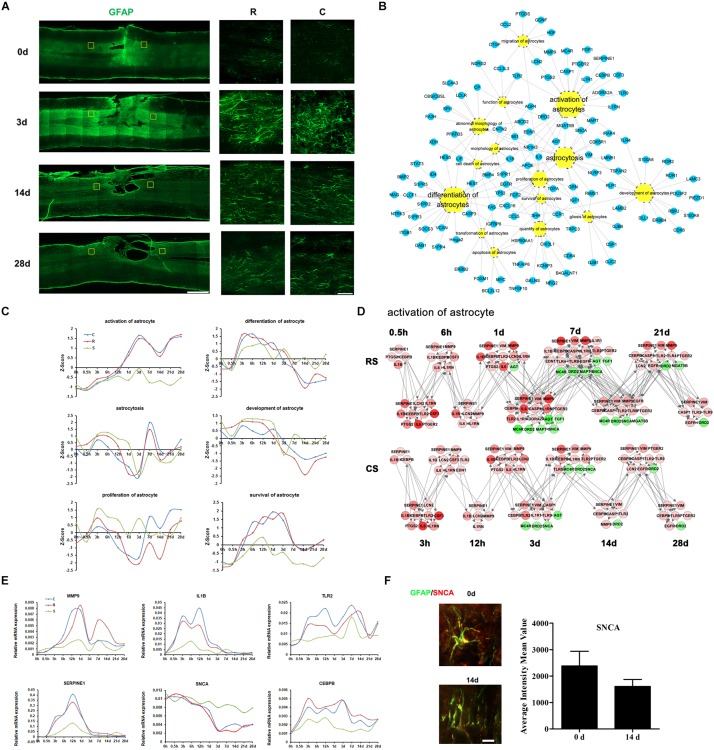
Molecular regulation of astrocyte responses to spinal cord injury (SCI). **(A)** Pathological changes in astrocytes following SCI as detected by GFAP antibody (green). Areas in the regions rostral (R) and caudal (C) to the injury site were selected (yellow squares) and magnified in the panels on the right. Scale bar: 1 mm (left panel), 50 μm (right panels). **(B)** Biological process enrichment analysis for the DEGs between injury and sham conditions that are involved in astrocyte processes. **(C)** The Z-Score of major biological processes in astrocytes over time. **(D)** Network maps of the changes in gene expression and regulation related to astrocyte activation following SCI. The red color means increased expression while the green color means decreased expression. The intensity of increase or decrease was illustrated by light/dark. The highly increased/decreased, the darker. **(E)** qRT-PCR validation of the expression over time of key genes involved in astrocyte activation. **(F)** Expression of SNCA (red) in the R region around the lesion at indicated time points. Astrocytes (green) expressing SNCA are shown in yellow. Scale bar: 10 μm.

We used GFAP as the marker of astrocyte, as shown in Figure 1A, astrocytes showed significantly larger cell bodies, hypertrophy of processes, and robust GFAP expression following SCI, indicating their reactive state. We then performed an IPA analysis to identify the DEGs involved in astrocytic biological processes (Figure 1B). Most of these DEGs were enriched in processes involved in astrocyte activation and differentiation. We analyzed the average expression profiles of the DEGs involved in vital astrocytic activities to obtain an overall view of the time-dependent changes in these processes. Six major biological processes of astrocytes (activation of astrocyte, differentiation of astrocyte, astrocytosis, development of astrocyte, proliferation of astrocyte, and survival of astrocyte) were shown, and we calculated the Z-scores for the expression trends of genes involved in these biological processes (Figure 1C). Compared with the sham group, astrocytes underwent survival, proliferation, activation, and astrocytosis sequentially after SCI, consistent with previous reports.

We then focused on astrocyte activation, which is an important biological process that involves changes in cell morphology and molecular expression in response to SCI. By IPA, we created a network map of gene expression and regulation during astrocyte activation. It showed that, after SCI, an increasing number of genes involved in astrocyte activation were upregulated in R vs. S until 7 days, although a set of genes was also downregulated from 1 day (Figure 1D and Supplementary Table S2). In the C vs. S group, the trends in gene expression were similar to those in R vs. S, except that the number of downregulated genes was smaller.

We selected six distinct changed genes for qRT-PCR validation (Figure 1E). Matrix metallopeptidase 9 (MMP9) and serpin family E member 1 (SERPINE1) reached the highest levels of expression at 12 h. Interleukin 1 beta (IL1b) was highly expressed from 3 h after SCI and returned to sham levels after 3 days. Toll-like receptor 2 (TLR2) and CCAAT enhancer binding protein beta (CEBPB) were also highly expressed following SCI. The expression of Synuclein Alpha (SNCA) decreased after injury, and this was reflected also in the immunofluorescence results (Figure 1F). SNCA is a pre-synaptic protein that self-associates into toxic oligomers (Burre, 2015). Our results indicate that SNCA may inhibit the activation of astrocytes in response to SCI.

### Molecular Regulation of Microglial Responses

**FIGURE 2:**
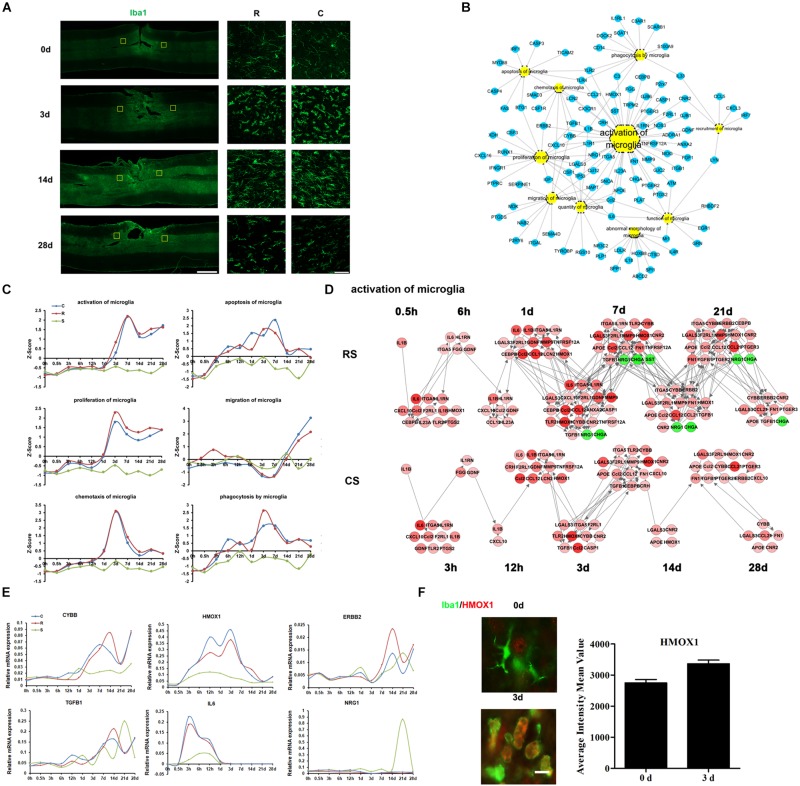
Molecular regulation of microglial responses to SCI. **(A)** Pathological changes in microglia and macrophages following SCI as detected by Iba1 antibody (green). Areas in the R and C to the injury site were selected (yellow squares) and magnified in the panels on the right. Scale bar: 1 mm (left panel), 50 μm (right panels). **(B)** Biological process enrichment analysis for the DEGs involved in microglial processes. **(C)** Average expression profiles of major biological processes in microglia. **(D)** Network maps of the changes in gene expression and regulation related to microglia activation following SCI. **(E)** qRT-PCR validation of key genes in microglial activation. **(F)** Expression of HMOX1 (red) in the R region around the lesion at indicated time points. Macrophage/microglia (green) expressing HMOX1 are shown in yellow. Scale bar: 10 μm.

The activation of microglia is a prominent hallmark of neuropathology following SCI, and is regulated by a variety of cytokines and chemokines. We carried out immunofluorescence staining for ionized calcium-binding adapter molecule 1 (IBA1), a marker of microglia and macrophages (which act to remove dead cells and cell debris). The staining showed that post-traumatic accumulation of microglia or invading macrophages was significantly increased at 3 days and the remained elevated up to 28 days after SCI (Figure 2A). The DEGs involved in microglia biological processes were enriched in activation of microglia (Figure 2B). The expression trends of DEGs in some enriched microglia biological processes were also shown in Figure 2C. The transcriptional profiles of the DEGs indicated a dynamic regulation of activated microglia, temporally controlled by multiple cytokines, chemokines, cell surface molecules, and proteases (Figures 2C,D and Supplementary Table S3). We observed that IL-6 was upregulated 3–12 h after SCI (Figures 2D,E). Cytochrome b-245 beta chain (CYBB), an enzyme system that generates reactive oxygen species (ROS) in microglia, was markedly upregulated following SCI. Erb-b2 receptor tyrosine kinase 2 (ERBB2) and transforming growth factor beta 1 (TGFB1) showed a slight increase. However, expression of anti-inflammatory heme oxygenase 1 (HMOX1), which is protective against oxidative stress in cells (Ghattas et al., 2002), increased substantially after SCI (Figures 2D,E); this supports the idea that activated microglia are both deleterious and beneficial to the functional outcome of the injured CNS. Immunofluorescence results confirmed the HMOX1 expression changes in microglia (Figure 2F).

### Molecular Regulation of Oligodendrocyte Responses

**FIGURE 3:**
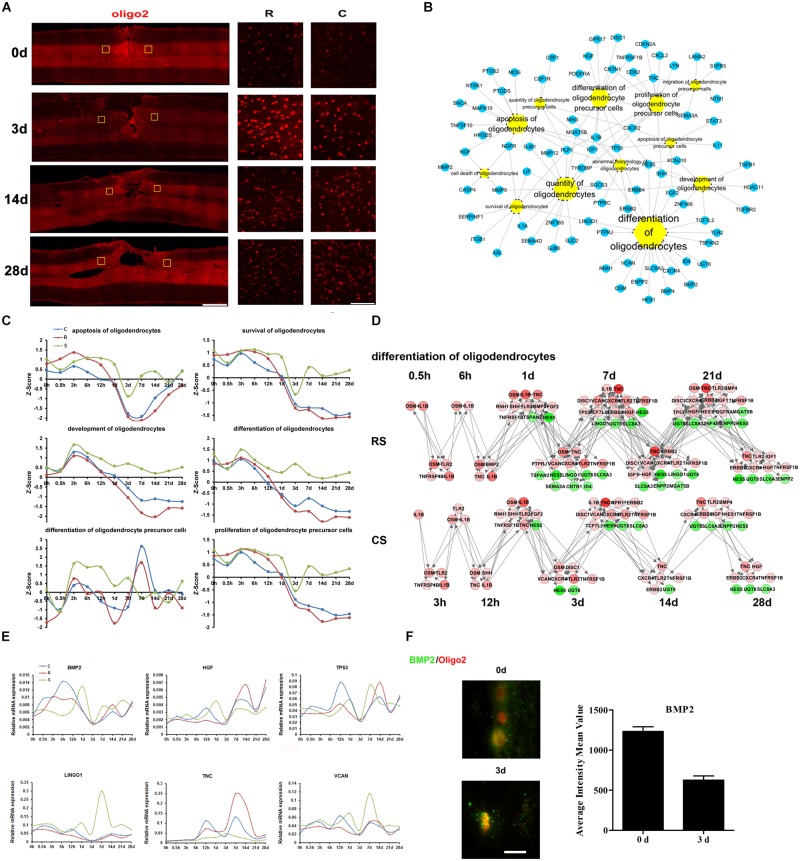
Molecular regulation of oligodendrocyte responses to SCI. **(A)** Pathological changes in oligodendrocytes following SCI as detected by oliog2 antibody (red). Areas in the R and C to the injury site were selected (yellow squares) and magnified in the panels on the right. Scale bar: 1 mm (left panel), 50 μm (right panels). **(B)** Biological process enrichment analysis for DEGs involved in oligodendrocyte processes. **(C)** Average expression profiles of major biological processes in oligodendrocytes. **(D)** Network maps of the changes in gene expression and regulation related to oligodendrocyte differentiation following SCI. **(E)** qRT-PCR validation of key genes in oligodendrocyte differentiation. **(F)** Expression of BMP2 (green) in the R region around the lesion at indicated time points. Oligodendrocyte (red) expressing BMP2 are shown in yellow. Scale bar: 10 μm.

Oligodendrocytes are a fundamental and unique cell type in the central nervous system and are responsible for myelinating axons (Kopp and Mendell, 2018). We used Oligo2 as a marker of oligodendrocytes. Immunofluorescence data showed a robust increase in oligodendrocytes at 3 days post-SCI, but the number of oligodendrocytes subsequently declined (Figure 3A). There seemed to be no notable differences in Oligo2 expression between the R and C sites.

Ingenuity pathway analysis analysis suggests that the DEGs associated with oligodendrocytes are mainly involved in oligodendrocyte differentiation, development, apoptosis, and survival (Figure 3B). We also calculated the Z-scores for the expression trends of genes involved in these biological processes (Figure 3C). The results showed that genes related to oligodendrocyte apoptosis, survival, development, and differentiation were upregulated prior to 1 day post-SCI and were downregulated at subsequent time points. Another interesting finding is that there was an upregulation of oligodendrocyte precursor cell differentiation in the S group from 3 h to 3 days, whereas this increase was delayed at following times in the R and C regions.

We then focused on oligodendrocyte differentiation, and created a network map of gene expression and regulation during this process (Figure 3D and Supplementary Table S4). From this, we selected distinct changed genes bone morphogenetic protein 2 (BMP2), hepatocyte growth factor (HGF), tumor protein p53 (TP53), leucine rich repeat and Ig domain containing 1 (LINGO1), tenascin C (TNC), and versican (VCAN) for qRT-PCR validation (Figure 3E). BMP signaling has been reported to suppress oligodendrocyte development (Cheng et al., 2007). In addition, the immunofluorescence results confirmed the changes in expression of the BMP2,a signaling molecule that regulates oligodendrogenesis (Figure 3F). These results indicate that BMP2 may play an important role in oligodendrocytes differentiation after SCI.

### Molecular and Cellular Immune Responses to Spinal Cord Injury

**FIGURE 4:**
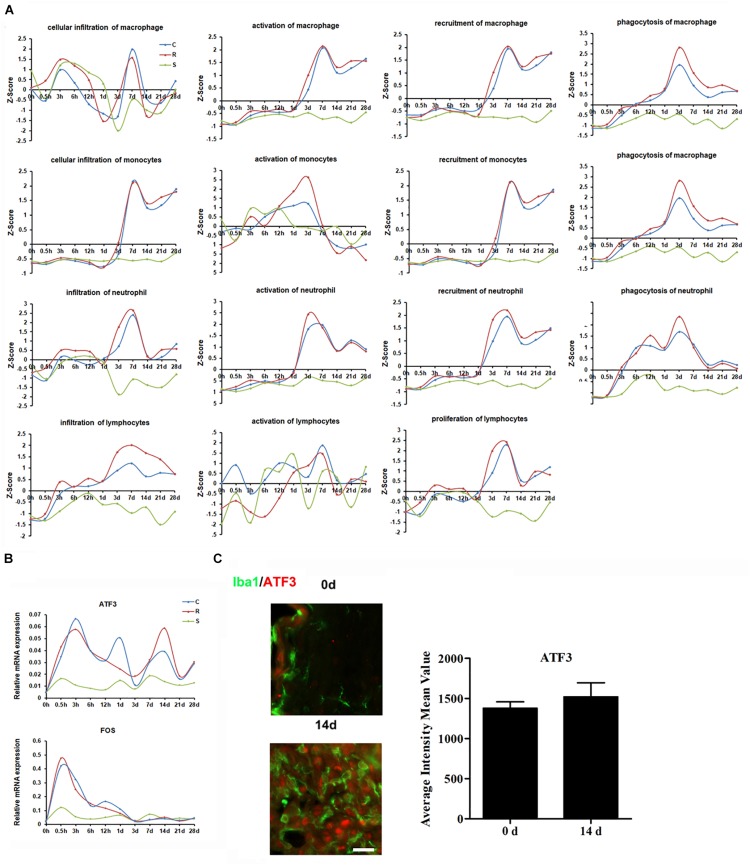
Molecular and cellular immune responses to SCI. **(A)** Average expression profiles of major biological processes involved in macrophages, monocytes, neutrophils, and lymphocytes. **(B)** qRT-PCR validation of key transcription factors in the immune system. **(C)** Expression of ATF3 (red) in the R region around the lesion at indicated time points. Macrophage/microglia (green) expressing ATF3 are shown in yellow. Scale bar: 10 μm.

Inflammation provoked by SCI can result in further tissue damage and neurodegeneration, hindering functional recovery. Z-score expression trends suggested that SCI triggers a sequential recruitment of neutrophils, monocytes, and lymphocytes to the lesion site (Figure 4A). Analysis of the transcriptional profiles indicated that cytokine IL-1β and other six chemokines (CCL3, CCL4, CCL5, CCL7, CXCL2, and CXCL3), were upregulated from 0.5 h post-SCI (Supplementary Figure S2c). It is interesting to note that several inflammation-related transcriptional factors, such as the members of the CCAAT/enhancer-binding protein (CEBP) family, ATF3, and RUNX proteins, were markedly upregulated and participate in the progression of the inflammatory responses (Supplementary Figures S2a,b). The functions of these transcriptional factors are deeply woven into immune processes; these transcriptional factors therefore have substantial potential to resolve inflammation in the injured spinal cord.

Next, we selected the distinct changed transcriptional factors ATF3 and FOS for qRT-PCR validation. The results show that the expression of both ATF3 and FOS increased following SCI, and that the elevated level of ATF3 was maintained until 28 days post-injury (Figure 4B). Immunostaining indicated that ATF3 was expressed in IBA1 positive cells (Figure 4C).

### Vascular System After Spinal Cord Injury

**FIGURE 5:**
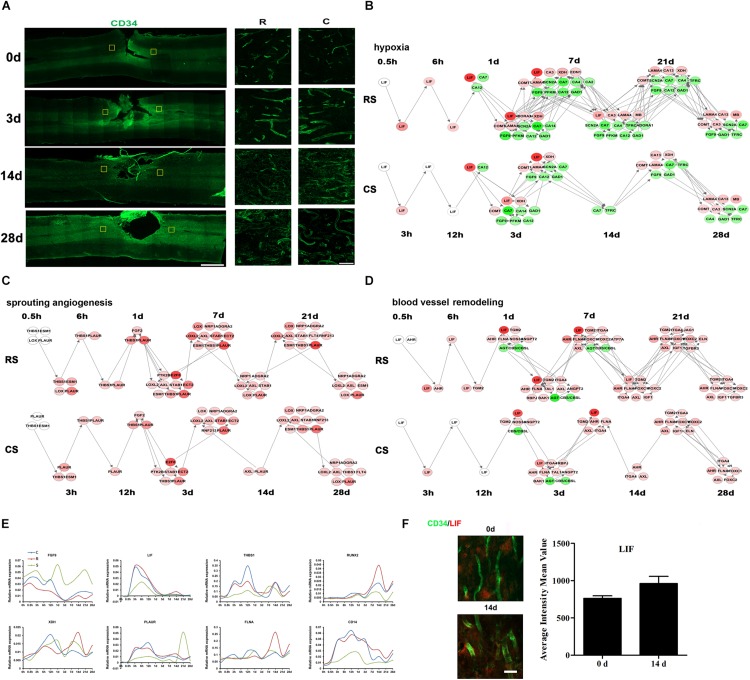
Vascular system after SCI. **(A)** Pathological changes in vascular system following SCI as detected by CD34 antibody (green). Partial areas in the regions rostral (R) and caudal (C) to the injury site were selected (yellow squares) and magnified in the panels on the right. Scale bar: 1 mm (left panel), 50 μm (right panel). Network maps of the changes in gene expression and regulation related to hypoxia **(B)**, sprouting angiogenesis **(C)**, and blood vessel remodeling **(D)** following SCI. **(E)** qRT-PCR validation of key genes in the vascular system. **(F)** Expression of LIF (red) in the R region around the lesion at indicated time points. Vascular system (green) expressing LIF are shown in yellow. Scale bar: 10 μm.

We used CD34 as a marker for blood vessels to detect vascular changes after SCI. The density of blood vessels was greater at 3 days after SCI than at 0 day, then decreased again by 14 days (Figure 5A). However, at 28 days, there appeared to be a slight increase in blood vessel density. Throughout, the density and intensity of blood vessels were weaker at the R site than at the C site. The IPA analysis showed that the DEGs associated with vascular endothelial cells (VECs) were mainly enriched in VEC migration, proliferation, adhesion, apoptosis, and activation pathways (Supplementary Figure S3a). We calculated the dynamic changes in these biological processes (Supplementary Figure S3b). In addition to these VEC processes, we also detected hypoxia, sprouting angiogenesis, and blood-vessel remodeling, which are three key events during angiogenesis after nerve injury. Dynamic networks for gene expression and regulation were mapped for hypoxia, sprouting angiogenesis, blood-vessel remodeling and VEC activation (Figures 5B–D and Supplementary Figure S3c, Tables S5–S7). We then performed qRT-PCR to validate several key genes involved in these processes, including FGF9, LIF, THBS1, RUNX2, XDH, PLAUR, FLNA, and CD14 (Figure 5E). Immunofluorescence results confirmed changes in LIF expression in blood vessels (Figure 5F).

## Discussion

**FIGURE 6:**
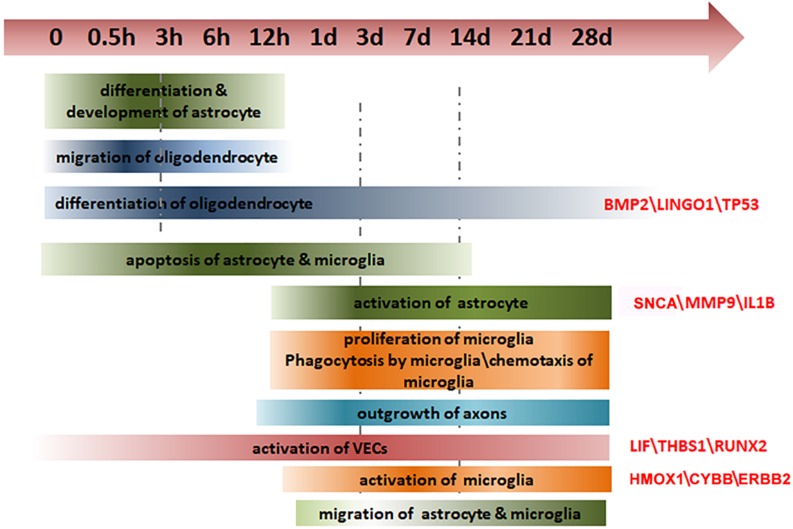
Summary of the changes in key genes and biological processes after SCI.

Spinal cord injury not only causes direct neurological dysfunction but is also accompanied by a cascade of pathophysiological events, such as inflammation, demyelination, and loss of blood supply, that contribute to a hostile microenvironment that inhibits nerve regeneration (Sinescu et al., 2010). Considering the poor outcome for functional recovery after SCI, it is crucial to obtain a thorough understanding of gene expression and molecular modulation following SCI. In this study, we hemisected the spinal cord in rats and measured the dynamic alterations in gene expression in the spinal cord regions R and C to the injury site, over 28 days after SCI. We examined the glial, immune, and vascular systems in detail. We identified the spatial and temporal characteristics of several pivotal genes in specific cell types, thus providing a global view of gene expression and molecular regulation following SCI (Figure 6).

Astrocytes are the major type of glial cell in the CNS, providing mechanical and metabolic support for neurons (Lukovic et al., 2015). After SCI, astrocytes become reactive and interact with other cells to form a glial scar, which is a barrier to axonal regeneration (Silver and Miller, 2004). However, it has also been suggested that astrocyte scar formation aids, rather than prevents, axon regeneration in the CNS (Anderson et al., 2016). Here, we identified scar formation around the injury site, and significant enrichment of activated astrocytes. SNCA is a 140-amino-acid natively unstructured brain protein that is particularly enriched in axon terminals (Burre, 2015). It is also the main constituent of the intraneuronal pathological aggregates known as Lewy bodies that are characteristically present in degenerating dopaminergic nigral neurons in patients with Parkinson’s disease (Poewe et al., 2017). Previous studies showed that spinal cord transection results in a significant downregulation of SNCA in the motor cortex of rats 3 days post-operatively (Wang et al., 2016). Here, we observed a clear decrease in SNCA expression in spinal astrocytes at 14 days post-SCI. These findings suggest that SNCA, as the main constituent of intraneuronal pathological aggregates, may inhibit glial scar formation, providing a novel starting point from which to explore the mechanisms underlying glial scar formation and their role in SCI.

Oligodendrocytes are the myelinating cells of the CNS, and 50% are lost by 24 h after nerve injury, leading to demyelination and impairments in axon conduction and neurological function (Zai and Wrathall, 2005; Church et al., 2016). Oligodendrocyte apoptosis continues for 2–3 weeks after the injury (Hesp et al., 2015). Neuron-glial antigen 2 (NG2)-positive oligodendrocyte progenitors subsequently proliferate and differentiate into new oligodendrocytes to restore the number of oligodendrocytes. In our study, BMP2 expression increased after injury, reaching a peak at 6 h and then decreasing. This pattern is consistent with previous reports (Hesp et al., 2015). Furthermore, Gao et al. (2006) showed that BMPs regulate retinal myelination during development. Together, these findings suggest that BMP2 participates in demyelination and remyelination after SCI.

The immune response begins almost immediately after mechanical injury and includes a constellation of events, including intraparenchymal hemorrhage and glial activation, with concomitant release of immune mediators (Pineau and Lacroix, 2007; Pineau et al., 2010). Microglia and macrophages play an important role in the maintenance of nervous system homeostasis, promoting phagocytosis, clearing debris, secreting proteases and elastase, and releasing ROS. After SCI, they can lead to pro-inflammatory activation and contribute to tissue damage (Francos-Quijorna et al., 2016). It was reported that after SCI, IL-1β, TNF were rapidly and transiently expressed in 15 mins and widely expressed in 45 min. From 3 to 24 h, IL-1β, TNF, and IL-6 was upregulated and disappeared by 2 days post-SCI with another expression wave for IL-1β and TNF at 14 days (Pineau and Lacroix, 2007). However, our data of cytokines expression showed some discrepancies with previous studies. We thought this might be due to different injury model and sample sections. In addition, most of these cytokine expression trends were obtained by RNA-seq without qRT-PCR validation. In future studies, we may confirm these cytokine expressions by qRT-PCR and also evaluate cytokine secretion post-SCI. Our IPA analysis indicated that neutrophils and microglia are the first immune cells to intervene after SCI and that they remain at the injury site for several days. Microglia, in particular, continue to maintain a high level of activation. Activation of monocytes and macrophages increased from 1 day after SCI. These cells release cytokines (such as TNF-α and IL-1b), nitric oxide, and leukotrienes, and have important phagocytic functions. Lymphocytes are the last immune cells to invade the lesion site, concomitantly with macrophages, and secrete cytokines in the lesion epicenter. These results are consistent with previous studies (Donnelly and Popovich, 2008; Neirinckx et al., 2014).

ATF3 is a member of the ATF/CREB family of transcription factors. Peripheral nerve injury induces high ATF3 expression in injured dorsal root ganglion neurons, and overexpression of ATF3 in cultured adult dorsal root ganglion neurons can enhance neurite outgrowth (Seijffers et al., 2007). In the CNS, ATF3 has also been reported to be upregulated after spinal cord and dorsal root injury (Huang et al., 2006). Our results show that ATF3 increased immediately after SCI, consistent with previous reports.

HMOX1 is a neuroprotective factor that is involved in signaling during the acute phase and has anti-inflammatory effects after SCI (Chen et al., 2013). HO-1-deficient macrophages showed reduced expression of IFN-β, which suggests that HMOX1 is a critical early mediator of the innate immune response (Tzima et al., 2009). Our results showed that HMOX1 gradually increases after SCI, peaking at 3 days post-SCI, consistent with previous findings (Liu et al., 2016) which also demonstrated the antinociceptive effect of HMOX1. These data suggest a neuroprotective role of microglial HMOX1 after SCI.

Together, our findings relating to the immune response after SCI will contribute to a more comprehensive understanding of how the immune system affects intraspinal and systemic processes after SCI.

Neurogenesis is closely associated with angiogenesis (the growth of new blood vessels from pre-existing ones) during neural tissue remodeling because immature neuronal migration and axonal sprouting can occur along neovessels in a lesion after nerve injury (Muramatsu et al., 2012; Yu et al., 2016). Angiogenesis has been observed at the epicenter of the gray matter lesion 3–7 days after SCI, which may minimize the lesion size and maintain neuronal viability (Benton et al., 2008). Consistent with this, we found that a number of genes involved in angiogenesis were dysregulated from 3 days after SCI. LIF, a member of the IL-6 family of cytokines, inhibits angiogenesis by modulating endothelial cell proliferation and migration (Pepper et al., 1995). In addition, LIF stimulates the differentiation and survival of oligodendrocyte precursors and prevents oligodendrocyte apoptosis (Kerr and Patterson, 2005). We detected high LIF expression during the early stage after SCI, which subsequently decreased substantially. Previous studies have shown that, 2 days after SCI, the density of blood vessels decreases and only residual blood vessels are observed at the injury site (Gong and Maquat, 2011). Angiogenesis is initiated 3–4 days after SCI and lasts for up to 1 week (Casella et al., 2002). Together, these findings suggest that LIF has a positive role in the regulation of oligodendrocyte survival and a negative role in angiogenesis.

Several therapeutic approaches have shown improvements in functional recovery after injury that are correlated with higher densities of blood vessels in the spinal cord (Glaser et al., 2006; Kaneko et al., 2006). The proper restoration of the blood-spinal cord barrier may help control the influx of inflammatory cells into the damaged spinal cord and direct the inflammatory response toward a regenerative path (Rocha et al., 2018). The key genes and biological processes related to angiogenesis identified in our work should contribute to the discovery of new and effective targets for SCI therapeutic interventions.

To date, there is no effective therapy for controlling the secondary injury following SCI, and the molecular mechanisms of SCI remain incompletely understood (Silva et al., 2014). The SCI transcriptome has been previously characterized by RNA-Seq (Chen et al., 2013), however, they used a contusive SCI model in mice and only collected samples at two time points (2 and 7 days after injury). In our research, we collected samples from a larger number of time points (0, 0.5, 3, 6, 12 h and 1, 3, 7, 14, 21, 28 days) in three groups (rostral region, caudal region, and sham control). We also adopted more comprehensive analysis and verification methods, thus providing a more detailed picture of the cell states and molecular expression modes in different cell types. Xiaoguang Li and colleagues discovered that neurotrophin-3-loaded chitosan provides an excellent microenvironment to facilitate axonal regrowth, neurogenesis, and functional recovery of completely transected spinal cords in rats (Duan et al., 2015). They used weighted gene coexpression network analysis to establish the gene modules/programs corresponding to various pathological events at different time points after SCI that was treated with neurotrophin-3-coupled chitosan biomaterial. By contrast, our work provides a view of the landscape of gene expression and regulation after SCI under normal pathological conditions, and thus bears more universal significance. However, though we compared R and C regions during our analysis, there seemed the difference between R and C was not of great significance compared to that of Sham control group, which was in consistence with previous research (Duan et al., 2015). In future, we might seek other analyses method to identify the differences between R and C region.

In summary, the present study demonstrates the dynamic changes in gene expression following SCI. We explored key biological processes in the microenvironment of the injury, and identified DEGs and events specific to the R or C regions. Our study provides a comprehensive description of the transcriptional changes and biological processes that occur after SCI, which we hope will facilitate the development of new molecular therapies for SCI.

## Materials and Methods

### Animal Surgery

A total of 200 adult male Sprague-Dawley rats (200–250 g) were used in this study. All animal procedures were performed in accordance with the Institutional Animal Care Guidelines of Nantong University and ethical approval was obtained from the Administration Committee of Experimental Animals, Jiangsu Province, China.

Spinal cord injury by left-side hemisection was performed as previously described (Christensen et al., 1996), with modifications. Rats were anesthetized with an intraperitoneal injection of mixed narcotics (85 mg/kg trichloroacetaldehyde monohydrate, 42 mg/kg magnesium sulfate, and 17 mg/kg sodium pentobarbital). After induction of anesthesia, a longitudinal incision was made, and a laminectomy was performed at vertebral segments T9-T10. The spinal cord was then hemisected at T9 on the right side by placing a 28-gauge needle dorsoventrally at the midline of the cord and pulling it laterally to ensure complete hemisection (without hemisection in sham surgery group). The fascia, musculature, and skin were closed using polyglycolic acid sutures for the aponeurotic plane and nylon thread for the skin. Throughout surgery, body temperature was maintained at 37°C with a heating blanket.

Upon recovery, the rats were returned to their home cages, with free access to food and water. Animals were group housed with littermates in a temperature controlled vivarium on a 12 h light-dark cycle. Distended bladders were emptied by manual massage on the lower abdomen twice per day until voluntary emptying resumed.

### RNA Sequencing

5-mm-long segments at the rostral, caudal region or sham surgery group were collected from 18 rats at each time point after SCI (0, 0.5, 3, 6, 12 h and 1, 3, 7, 14, 21, 28 days). The segments of six rats in each time points were mix together for RNA extraction and RNA sequencing. Thus each time points we have three repeated samples for analysis. Total RNA of these samples were extracted with a mirVana miRNA Isolation Kit (Ambion) following the manufacturer’s protocol. RNA integrity was evaluated using an Agilent 2100 Bioanalyzer (Agilent Technologies, Santa Clara, CA, United States). Samples with an RNA Integrity Number (RIN) ≥ 7 were subjected to further analysis. Libraries were constructed using TruSeq Stranded Total RNA with Ribo-Zero Gold, according to the manufacturer’s instructions. These libraries were subsequently sequenced on the Illumina HiSeq^TM^ 2500 sequencing platform and 150 bp/125 bp paired-end reads were generated (Shanghai OE Biotech, Co., Ltd.).

All data can be viewed in NODE^1^ by pasting the accession (OEP000369) into the text search box or through the URL: http://www.biosino.org/node/project/detail/OEP000369.

### Differentially Expressed Genes

Raw data (raw reads) were processed using the NGS QC Toolkit (Patel and Jain, 2012). The reads that contained poly-N sequences and low-quality reads were removed to obtain clean reads. Fragments Per Kilobase of transcript per Million fragments mapped (FPKM) and the read count value of each transcript (protein_coding) were calculated in Bowtie 2 (Langmead and Salzberg, 2012) and eXpress (Roberts and Pachter, 2013). DEGs were identified with the DESeq (2012) functions estimate SizeFactors and nbinomTest. *P* < 0.05 and FoldChange > 2 or FoldChange < 0.5 were set as the thresholds for significantly differential expression.

### Ingenuity Pathway Analysis (IPA)

The online software package IPA^2^ (Ingenuity Systems, Redwood City, CA, United States) was used to identify the biological processes and gene networks for the DEGs. (i) Enriched biological processes according to cell type. We searched diseases and functions associated with specific cell types (e.g., astrocytes, microglia, oligodendrocytes) and identified the genes involved in these diseases and functions. We then filtered these genes with the DEGs, and the overlapped genes coverage genes were constructed into a network according to their relevant functions. (ii) Average expression profiles of the major biological processes. The average expressions of major biological processes were calculated as described previously (Chen et al., 2003; Viader et al., 2011). (iii) The regulation network between DEGs in certain biological processes. We used IPA analyses to get the regulation relationships between genes in certain processes and construct the regulation networks at different time points. We then selected genes for qPCR according to the regulation network of certain biological processes. There are two criterions for the selection: (1) The expression of gene following SCI. Genes with deep red or deep green in the regulation networks suggesting that this gene has a great expression difference compared with sham group. (2) The relationships of gene with other genes in the regulation networks. Genes with more relationships in the regulation network suggesting that this gene was a key node in the regulation network. We then selected genes meet these two criterions for qPCR validation.

### Quantitative Reverse Transcription Polymerase Chain Reaction

RNA was extracted using TRIzol reagent (Invitrogen), and qRT-PCR was performed according to the manufacturer’s instructions. In brief, cDNA was synthesized by reverse transcription (Takara), then quantified using SYBR Premix Ex Taq II (Takara) on a QuantStudio 6 Flex instrument (Applied Biosystems). All assays were performed in triplicate, and the results were normalized to β-actin expression. The primers are listed in Supplementary Table S8.

### Immunofluorescence

Rats used for immunofluorescent studies were euthanized by intraperitoneal injection of mixed narcotics and transcardially perfused with 4% paraformaldehyde. A 10-mm-long sample of spinal cord running from the rostral (R) region of the injury site to the caudal (C) region of the injury site was collected at the indicated times after surgery (*n* = 3 per timepoint). All tissues were post-fixed for an additional 6 h before being transferred to 30% sucrose and longitudinally cryo-sectioned at 40 μm and direct mounted on slides. Slide-mounted sections were incubated in primary antibodies at 4°C for 24 h, followed by further reaction with the secondary antibody at room temperature for 1 h. Finally, the sections were observed and photographed under fluorescence microscopy (AxioImager M2, Zeiss). The antibodies used are listed in Supplementary Table S9.

### Statistical Analysis

All data are presented as the means ± SEM. The data of qRT-PCR were compared using Student’s *t*-test in Prism 5 (GraphPad, San Diego, CA, United States). *P* < 0.05 was considered statistically significant.

## Data Availability Statement

The datasets generated for this study can be found in the all data can be viewed in NODE (http://www.biosino.org/node) by pasting the accession (OEP000369) into the text search box or through the URL: http://www.biosino.org/node/project/detail/OEP000369.

## Ethics Statement

The animal study was reviewed and approved by all animal procedures were performed in accordance with the Institutional Animal Care Guidelines of Nantong University and ethical approval was obtained from the Administration Committee of Experimental Animals, Jiangsu Province, China.

## Author Contributions

XG, FD, and BY designed the experiments and supervised the project. CY, YaW, SM, RW, WF, and YC performed the experiments. BY, CY, and SM analyzed the results. YoW, DL, CX, and JY gave suggestions for the experiments and analysis. CY, SM, and YoW wrote the manuscript. BY and CY revised the manuscript.

## Conflict of Interest

The authors declare that the research was conducted in the absence of any commercial or financial relationships that could be construed as a potential conflict of interest.

## Footnotes

1 http://www.biosino.org/node

2 http://ingenuity.com/index.html
