# Supplementary material for: The Landscape of Gene Expression and Molecular Regulation Following Spinal Cord Hemisection in Rats
Source: Front Mol Neurosci. 2019 Nov 22;12:287. doi: 10.3389/fnmol.2019.00287 (PMC6883948; doi:10.3389/fnmol.2019.00287)
Supplement: Supplementary file 10 [file Table_10.DOC]

**Supplementary Figures**

**
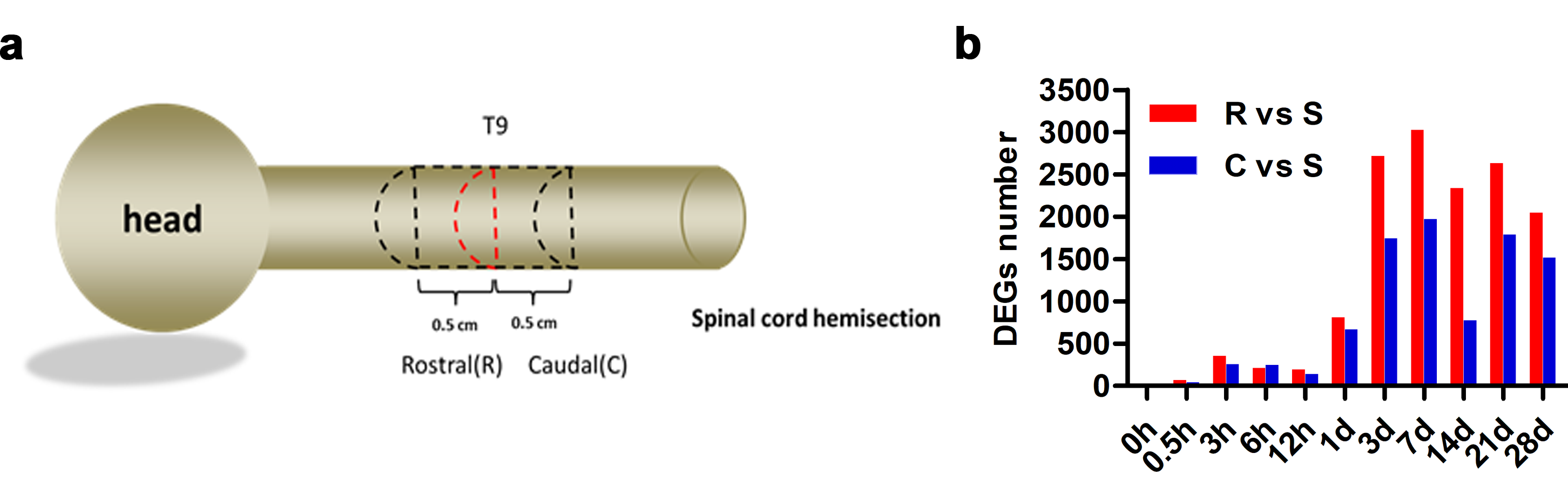
**

## Figure S1. DEGs following spinal cord hemisection in rats.

**a** Illustration of spinal cord hemisection surgery. **b** DEGs in rostral (R) and caudal (C) regions compared with the sham (S) group.


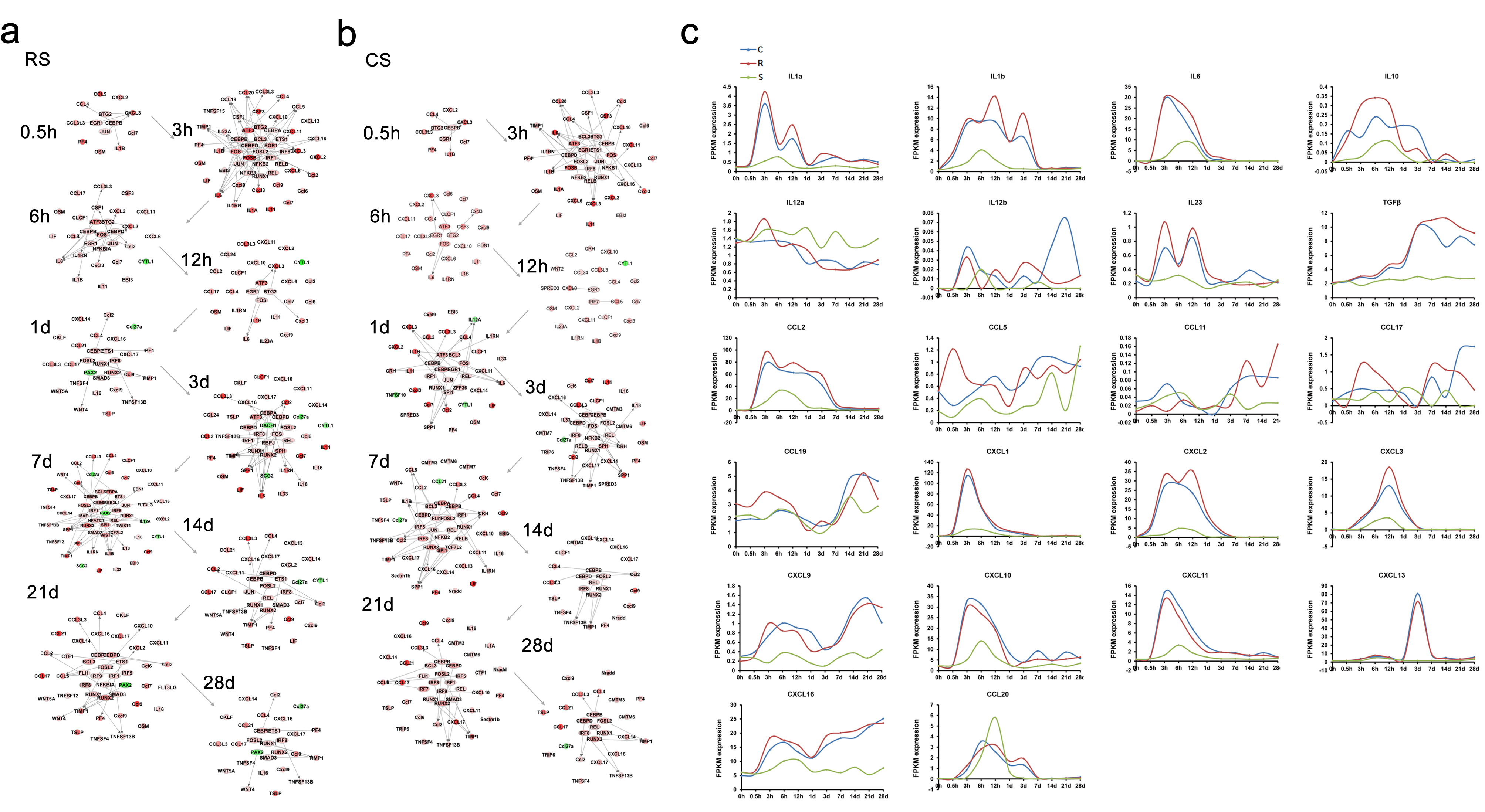


## Figure S2. Molecular and cellular immune responses to SCI. a-b

Network maps of the changes in gene expression and regulation in cytokines and their transcription factors following SCI in a) the R region and b) the C region. The circles mean dysregulated cytokines and the ellipses mean transcription factors regulating these cytokines. **c** FPKM expression of differentially expressed cytokines following SCI. We have selected some key cytokines and explored their expression alteration.


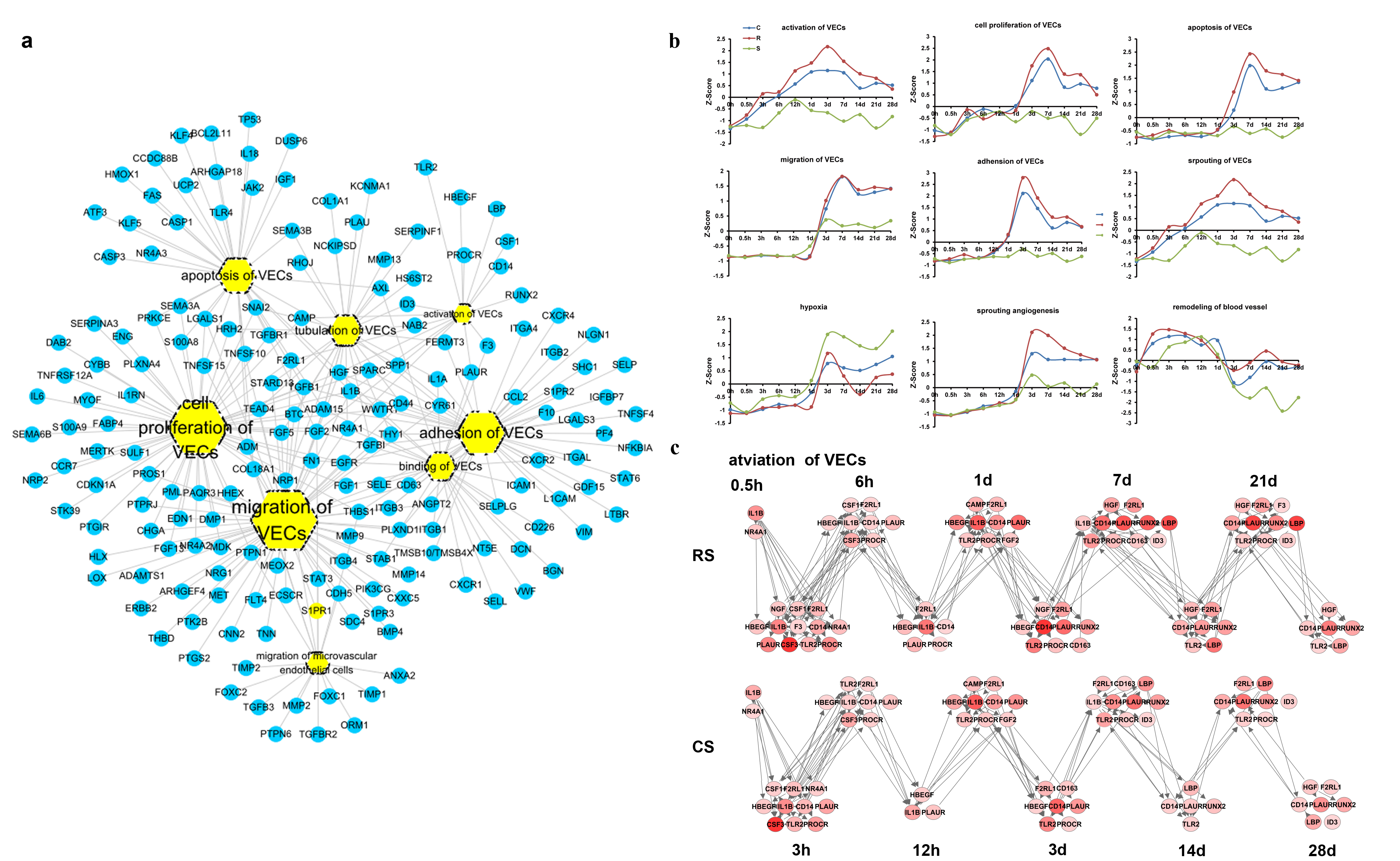


## Figure S3. Vascular endothelial cell response to SCI.

**a** Biological process enrichment analysis of DEGs involved in VECs. **b** Average expression profiles of major biological processes in VECs. **c** Network of the changes in gene expression and regulation in VEC activation following SCI.
